# How physical exercise impacts academic burnout in college students: The mediating effects of self-efficacy and resilience

**DOI:** 10.3389/fpsyg.2022.964169

**Published:** 2022-11-11

**Authors:** Kai Chen, Feiyang Liu, Liu Mou, Peiting Zhao, Liya Guo

**Affiliations:** ^1^School of Physical Education, Southwest University, Chongqing, China; ^2^Department of Physical Education, Chongqing University of Technology, Chongqing, China

**Keywords:** academic burnout, college, physical exercise, resilience, self-efficacy

## Abstract

**Background and aims:**

Academic burnout is harmful to college students, their institutions of learning, and society at large. While research has shown that physical exercise may be associated with reduced academic burnout, the underlying mechanisms require further exploration. This study explored the relationship between physical exercise and academic burnout in a sample of college students, with a focus on the serial mediating roles of self-efficacy and resilience.

**Methods:**

This study adopted a cross-sectional survey approach among a sample of undergraduate college students in China. We recruited 1,270 participants in the second half of the 2021–2022 academic year (476 men and 794 women), all of whom completed questionnaires containing the Physical Activity Rating Scale, Academic Burnout Scale for College Students, 10-item General Self-Efficacy Scale, and 25-item Connor-Davidson Resilience Scale. We then subjected the collected data to a series of statistical analyses.

**Results and conclusion:**

Physical exercise was significantly and negatively associated with academic burnout and its three subfactors (i.e., emotional exhaustion, improper behavior, and low personal achievement). Participants in the high physical exercise group showed lower levels of academic burnout than those in the moderate and low physical exercise groups. Finally, our serial mediation model showed that physical exercise had a significant direct effect on academic burnout (β = −0.1104, 95% CI = [−0.1421, −0.0791]) in addition to significant indirect effects on academic burnout *via* self-efficacy and resilience (β = −0.0802, 95% CI = [−0.1088, −0.0527]); the more exercise participation, the lower the academic burnout among college students. These findings suggest that physical exercise is an important interventional target when aiming to reduce academic burnout.

## Introduction

Academic burnout is an extreme form of maladjustment to school education that negatively impacts students at all academic levels (Romano et al., 2021). It is more specifically defined as a psychological and behavioral disorder that occurs when students are disinterested in learning or lack the motivation to learn, and may thus emerge as emotional exhaustion, disengagement from learning, and low personal achievement (Schaufeli et al., 2002; Lian et al., 2005). As a result, academic burnout can seriously impact academic performance, physical health, and mental health, and is even known as a precursor to depression (May et al., 2015; Lee and Lee, 2018; Aghajani Liasi et al., 2021). Moreover, the condition is particularly problematic for college students, as it may eventually lead to professional burnout, which can threaten the healthy development of employment across society (Robins et al., 2018). Taken together, these issues highlight the need for continued research aimed at preventing and alleviating academic burnout, thus avoiding damage at the individual, institutional, and societal levels.

Previous studies have referred to two strategic goals to prevent or alleviate academic burnout in college students. One is to develop positive individual qualities, such as optimism and grit (Vizoso-Gomez et al., 2019; Ozhan, 2021), while the other is to enhance resources that are provided by the family, school, and society (Salmela-Aro and Upadyaya, 2017; Trigueros et al., 2020; Ye et al., 2021). Although these studies widely discussed the relationships between stress, negative emotions, maladjustment, and academic burnout (Lin and Huang, 2014; Merhi et al., 2018; Vinter et al., 2021), none have simultaneously examined the relationships among physical exercise, self-efficacy, resilience, and academic burnout, which are thus focal points in this study.

The term “physical exercise” refers to physical activity that is accomplished at a certain intensity, frequency, and duration, with the aim of benefitting physical and mental health (Pan et al., 2021). Such engagement not only helps individuals achieve positive changes in physicality and mood, but also reduces their physiological sensitivity to stress (Sani et al., 2016; Gerber et al., 2017; Mikkelsen et al., 2017) while increasing academic performance and facilitating social adjustment (Ji and Zheng, 2021; Wunsch et al., 2021). As each of these gains can reduce academic burnout, some studies have directly investigated the impacts of physical exercise. For example, one study found that vocational students who completed the recommended level of moderate-to-vigorous physical activity reported fewer symptoms of burnout (Gerber et al., 2015), while another study found that only vigorous physical activity was associated with reduced burnout (Elliot et al., 2015). There is also evidence that suggests that signs of burnout among Brazilian university students are significantly and negatively associated with physical activity (De Souza et al., 2021). However, there is still a lack of data on these issues in the college student population, with no such evidence in the Chinese context. As such, this study examined how physical exercise affected academic burnout in a sample of Chinese college students, as outlined in the following hypothesis:

*H1:* Physical exercise would be negatively associated with academic burnout in college students.

Although it is possible that physical exercise can directly reduce academic burnout, it is important to consider whether other variables mediate the process in which this occurs. Indeed, previous research has concluded that self-efficacy plays an important mediating role in the relationship between physical exercise and mental health in college students (Jiang et al., 2017). More specifically, self-efficacy refers to an individual’s perceptions or beliefs about their own ability to act appropriately when faced with difficult situations or new circumstances (Schwarzer et al., 1997). Under the relevant theoretical framework (Bandura et al., 1999), good physical and mental states motivate individuals to establish self-efficacy, which may therefore be enhanced through physical exercise, given its positive effects on both physical and mental health. For example, there is evidence that college students who regularly participate in physical exercise have higher levels of self-efficacy (Joseph et al., 2014). At the same time, self-efficacy affects individual thinking patterns and sensory responses (Bandura et al., 1999). In this regard, students with high self-efficacy are typically more able to choose appropriate strategies in times of failure or academic stress, thus reducing the intensity of any academic burnout symptoms (Rahmati, 2015; Luthfia et al., 2021). We therefore posited the following hypothesis:

*H2:* Self-efficacy would mediate the relationship between physical exercise and academic burnout in college students.

Resilience is often mentioned in conjunction with self-efficacy when discussing how individuals can improve their ability to cope with adversity. In this context, resilience refers to the characteristics, processes, and outcomes by which individuals are able to cope with adversity and eventually return to a positive state (Connor and Davidson, 2003; Fletcher and Sarkar, 2013; Robertson et al., 2015). There is existing evidence that resilience may play an important role in limiting academic burnout, as it is negatively associated with emotional exhaustion and moderates the relationship between academic burnout and perceived mental health in college students (García-Izquierdo et al., 2018). Meanwhile, physical exercise appears to be a significant predictor of resilience, as it strengthens an individual’s physical structure and thus provides support during psychological struggles in times of difficulty (Sahin et al., 2012; Seçer, and Çakmak yildizhan, 2020). Based on this, we posited the following hypothesis:

*H3:* resilience would mediate the relationship between physical exercise and academic burnout in college students.

Although the literature suggests that self-efficacy and resilience independently influence the relationship between physical exercise and academic burnout, these factors may also be linked together. Indeed, many studies have focused on this connection. For example, self-efficacy positively predicts resilience, it is an important characteristic in differentiating resilience levels among adolescent students (Hamill, 2003). There is also evidence that self-efficacy both promotes the development of resilience and mediates the effects of other positive factors on resilience (Sabouripour et al., 2021). In addition, increased resilience helps college students academic tasks, engage in professional practice, and develop well-being, thereby reducing the risk of academic burnout (Chow et al., 2018). As such, it is reasonable to suggest that physical exercise can influence resilience by affecting self-efficacy, which then affects academic burnout, as outlined in the following hypothesis:

*H4:* Self-efficacy and resilience would play serial mediating roles in the relationship between physical exercise and academic burnout in college students.

Demographic variables (e.g., gender, age, grade level, degree subject, academic year) have been of interest in prior research on academic burnout (Aguayo et al., 2019). In particular, gender has received attention because there are no consistent results from the academic community regarding whether there are gender-related differences in academic burnout among college students (Onuoha and Akintola, 2016). Therefore, this study included gender variables in the relationship between physical exercise and academic burnout. In sum, we established a hypothetical serial mediation model with physical exercise set as the independent variable, academic burnout set as the dependent variable, and self-efficacy/resilience set as mediating variables. Using this framework, we investigated various effects and mechanisms in the relationship between physical exercise and academic burnout, thus providing a practical basis for solving relevant problems. In this regard, we hope that our findings will motivate college campus administrators to help students adopt strategies that promote physical exercise.

## Materials and methods

### Participants and procedure

We used a cross-sectional survey method to collect data from university students. In general, under China’s key university system, differences have been noted in the mental health changes experienced by students attending key and regular universities (Xin et al., 2012; Feng, 2022). We were aware that this may lead to a difference in the prevalence of academic burnout among students at these different types of universities. To take this into account in the sample selection process, we conducted surveys at one key university and one regular university in Chongqing, and four physical education course classes per grade in each university were randomly selected. In March 2022, when all participants were in the second half of the 2021–2022 academic year, we sent requests to the lecturers of the prospective participants’ classes, inviting the lecturers to assist us in recruiting participants from their classes. We sent detailed information about the purpose of the study to the lecturers who relayed this to the students in their classes.

A total of 1,400 undergraduate students agreed to participate in our study and 1,345 questionnaires were returned, with a response rate of 96.07%. In each case, the questionnaires completed by the participants took approximately 10 min to finish. After we excluded incomplete questionnaires and outliers, the final study included a sample of 1,270 (94.42%), 638 from key universities and 632 from regular universities (476 men and 794 women; 403 social science majors, 867 natural science majors; 338 freshmen, 333 sophomores, 308 juniors, and 291 seniors). All participants provided informed written consent, and were compensated with 2 RMB. The survey protocol was authorized by the Southwestern University Human Ethics Committee (approval no. 20220304C1), and adhered to the Helsinki Declaration of Ethical Standards.

### Materials

#### Physical exercise

We investigated physical exercise *via* the revised Physical Activity Rating Scale (PARS-3; Liang, 1994), which has been widely used to measure the level of physical exercise in the Chinese population and shown to have sufficient validity (Lin and Chai, 2019). The scale has three items that measure exercise intensity, exercise frequency, and single exercise time respectively, each of which is divided into five levels and scored on a scale ranging from 1 to 5. According to Liang’s recommendations, physical exercise score = exercise intensity score _*_ (exercise time score − 1) _*_ exercise frequency score. The score interval is from 0 to 100 points and can be divided into three criteria to describe the different levels of physical exercise: low physical exercise ≤19 points, medium physical exercise 20–42 points, and high physical exercise ≥43 points (Liang, 1994). As the scale could not be tested for consistency, we arranged the three items at the beginning and end of each questionnaire to test for reliability, and then removed all unreliable questionnaires (e.g., those containing inconsistent information).

#### Academic burnout

We measured academic burnout *via* the Learning Burnout Scale for College Students (Lian et al., 2005), which assesses three factors, including emotional exhaustion (“I felt exhausted after learning for a whole day”), improper behavior (“I rarely organized my study time”), and low personal achievement (“I have the ability to get my bachelor’s degree”; reverse scored). The scale contains 20 total items that are rated on a 5-point Likert-type scale ranging from 1 (*totally disagree*) to 5 (*totally agree*). Total scores may therefore range from 20 to 100, with higher scores indicating higher academic burnout. In this study, the scale was internally consistent based on a Cronbach’s alpha value of 0.894.

#### Self-efficacy

We measured self-efficacy *via* the Chinese version of the General Self-Efficacy Scale (GSES; Zhang and Schwarzer, 1995), which focuses on self-confidence when facing difficulty (e.g., “I can always find a solution to a problem when I am faced with it”). The GSES contains 10 total items that are rated on a 4-point Likert-type scale ranging from 1 (*totally disagree*) to 4 (*totally agree*), with higher scores indicating higher self-efficacy. In this study, the Chinese version of the GSES was internally consistent based on a Cronbach’s alpha value of 0.917.

#### Resilience

We measured resilience *via* the Chinese version of the 25-item Connor-Davidson Resilience Scale (CD-RISC; Yu and Zhang, 2005), which contains three dimensions, including toughness (“I do not let failure get me down easily”), strength (“I’m proud of my accomplishments”), and optimism (“I try to look at the humorous side of things when faced with a problem”). The items are rated on a 5-point Likert scale ranging from 1 (*totally disagree*) to 5 (*totally agree*). In this study, the Chinese version of the CD-RISC was internally consistent based on a Cronbach’s alpha value of 0.956.

### Statistical analyses

We conducted our analyses using IBM SPSS 26.0 and Process 3.5 macros for SPSS. First, we tested the normality of the sample data and then analyzed the means and standard deviations of each variable to determine if they were demographically different. Second, we tested the relationships between all variables *via* Pearson’s bi-variate correlation analysis. Third, we set physical exercise as a categorical variable (low, medium, and high) according to the criteria of the PARS-3 scale (Liang, 1994), and then analyzed academic burnout for each genders in these three physical exercise groups using ANOVA and independent samples t-tests. Finally, we used Model 6 of the Process 3.5 macros for SPSS to test the multiple mediation model. We applied bootstrapping with coefficients estimated from 5,000 bootstraps to compute the direct effect and indirect effect. In cases where the 95% bias-corrected confidence interval (CI) did not include zero, the effect was significant (Hayes, 2017). We verified the efficacy of each analysis based on the effect sizes of the respective results using the G*Power 3.1 post-hoc power analysis (Faul et al., 2009).

As recommended in previous research (Podsakoff et al., 2003), we used the Harman’s single-factor test to investigate the potential for common method bias. The unrotated principal component factor analysis showed that there were seven factors with eigenvalues greater than 1; the first factor explained 36.17% of the variance. Thus, the study questionnaire was not seriously affected by common method bias.

## Results

### The relationships between physical exercise, self-efficacy, resilience, and academic burnout

The absolute values of skewness for all four variables were less than 2, while the absolute values of kurtosis were less than 7; thus, the data were approximately normally distributed (Kline, 2015). Table 1 shows the means (standard deviations) of physical exercise, self-efficacy, resilience, and academic burnout in the study sample, as well as their differences in demographic variables. All variables showed significant gender-related differences except academic burnout; among college students of different grades and different majors, there were no significant differences in all variables except physical exercise. Table 2 shows the results of the correlation between all variables. We found significant positive correlations between physical exercise, self-efficacy, and resilience. Academic burnout and its three subfactors were significantly and negatively related to physical exercise, self-efficacy, and resilience. In the relationships between physical exercise and the three subfactors of academic burnout, low personal achievement had the highest correlation, respectively followed by emotional exhaustion and improper behavior. In addition, low achievement was strongly correlated with self-efficacy and resilience, while improper behavior and emotional exhaustion showed relatively weak correlations with these factors. The power value for this analysis was 1 (ρ H1_min_ = −0.24, α = 0.01).

**Table 1:** Mean (SD) of physical exercise, self-efficacy, resilience, and academic burnout and the differences between these variables and demographic variables.

Variables		Physical exercise	Self-efficacy	Resilience	Academic burnout
Total	Mean(SD)	21.76(22.06)	28.76(5.21)	93.07(15.03)	54.58(11.97)
	Skewness	1.14	−0.15	−0.77	−0.20
	Kurtosis	0.32	0.13	1.76	−0.46
Gender	Male	29.99(24.49)	29.92(5.55)	95.32(16.60)	54.21(13.09)
	Female	16.82(18.82)	28.07(4.86)	91.73(13.84)	54.80(11.24)
	t value	10.08^***^	6.03^***^	3.97^***^	−0.82
Grade	Freshman	16.93(16.81)	28.59(4.72)	94.46(14.20)	54.91(10.85)
	Sophomore	19.68(20.64)	28.34(4.64)	93.53(13.39)	53.78(11.21)
	Junior	23.15(23.11)	28.82(5.50)	92.03(15.91)	55.56(12.19)
	Senior	28.27(25.91)	29.41(5.95)	92.04(16.63)	54.08(13.74)
	*F* value	15.76^***^	2.37	2.02	1.43
Major	Social sci.	24.04(24.52)	28.87(5.68)	92.25(17.11)	53.65(12.40)
	Natural sci.	20.70(20.75)	28.71(4.98)	93.45(13.95)	55.01(11.74)
	t value	2.37^*^	0.48	−1.23	−1.88

*** *p*<0.001.

* *p*<0.05.

**Table 2:** Correlation coefficients between the study variables.

Variables	1	2	3	4	5	6
1 Physical exercise	1					
2 Self-efficacy	0.43^**^	1				
3 Resilience	0.37^**^	0.78^**^	1			
4 Academic burnout	−0.41^**^	−0.54^**^	−0.53^**^	1		
5 Emotional exhaustion	−0.33^**^	−0.31^**^	−0.24^**^	0.89^**^	1	
6 Improper behavior	−0.28^**^	−0.40^**^	−0.42^**^	0.88^**^	0.71^**^	1
7 Low personal achievement	−0.38^**^	−0.72^**^	−0.77^**^	0.62^**^	0.25^**^	0.43^**^

** *p* < 0.01.

To further explore the effects of different physical exercises on academic burnout, we compared the mean value of academic burnout in the physical exercise (low, medium, and high) groups, and tested for differences in this effect across genders. As shown in Table 3, there were significant differences in academic burnout among total, male, and female college students across the different physical exercise groups. Post-hoc tests showed statistically significant differences between the groups except for the medium exercise group and the high exercise group of male students. The mean values of academic burnout are arranged from low to high, as follows: high physical exercise group < medium physical exercise group < low physical exercise group. There was a significant gender-based difference in academic burnout between the low and high exercise groups. The power value for the above analysis was 1 (effect size f_min_ = 1.038, α = 0.01).

**Table 3:** Comparison of mean (SD) of academic burnout among college students in different physical exercise groups.

Variables	Gender			Total
	Male	Female	t value	Mean(SD)
Low physical exercise	59.93(10.95)	57.62(9.93)	2.82^**^	58.25(10.26)
Medium physical exercise	50.80(12.44)	50.12(11.04)	0.44	50.44(11.69)
High physical exercise	48.66(13.10)	44.98(10.95)	2.45^*^	47.18(12.39)
F value	45.70^***^	74.86^***^	-	118.42^***^
Post-hoc	|L − M|^***^|L − H|^***^	|L − M|^***^|L − H|^***^|M − H|^**^	-	|L − M|^***^|L − H|^***^|M − H|^***^

Independent variable: academic burnout. |L − M|: Low physical exercise and Medium physical exercise. |L − H|: Low physical exercise and High physical exercise. |M − H|: Medium physical exercise and High physical exercise.

*** *p*<0.001.

** *p*<0.01.

* *p*<0.05.

### Mediated model: The effects of physical exercise on academic burnout

**Figure 1:**
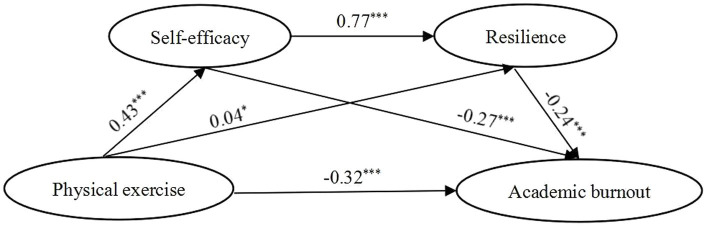
Model showing the mediating roles of self-efficacy and resilience in the relationship between physical exercise and academic burnout (Total sample). ***p < 0.001, *p < 0.05; significant standardized regression coefficient.

**Figure 2:**
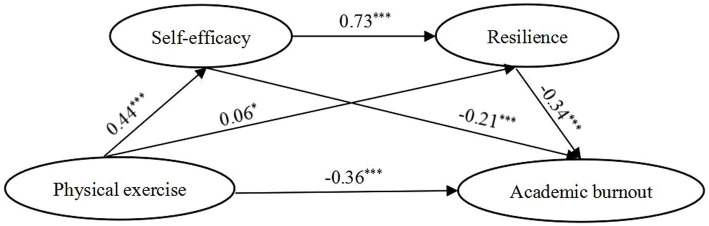
Model showing the mediating roles of self-efficacy and resilience in the relationship between physical exercise and academic burnout (Female sample). ***p < 0.001, *p < 0.05; significant standardized regression coefficient.

**Figure 3:**
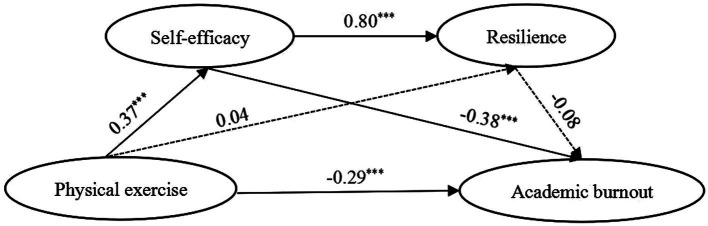
Model showing the mediating roles of self-efficacy and resilience in the relationship between physical exercise and academic burnout (Male sample). ***p < 0.001; significant standardized regression coefficient.

With physical exercise set as the independent variable, academic burnout set as the dependent variable, and self-efficacy/resilience set as mediating variables, we tested the serial mediation model for the total sample and across genders sample *via* Process 3.5. In both the overall sample and female sample (Figures 1–2; Table 4), physical exercise not only had a significant negative direct effect on academic burnout, but also had a significant negative indirect effect on academic burnout through three pathways: indirect path 1 (physical exercise → self-efficacy → academic burnout), indirect path 2 (physical exercise → resilience → academic burnout), indirect path 3 (physical exercise → self-efficacy → resilience → academic burnout). In the male sample (Figure 3; Table 4), physical exercise had a significant negative direct effect on academic burnout and a significant negative indirect effect on academic burnout only through self-efficacy. The power value was 1 (α = 0.05) for the direct path and indirect path 1; the power value of indirect path 2 was less than 0.296 (α = 0.5).

**Table 4:** The path and standardized indirect/direct effects of physical exercise on academic burnout in college students.

Path	Total			Male			Female		
	Effect size	SE	95%CI	Effect size	SE	95%CI	Effect size	SE	95%CI
Direct effect	−0.1104	0.0160	−0.1421, −0.0791	−0.1187	0.0240	−0.1654, −0.0711	−0.1369	0.0202	−0.1762, −0.0978
Total indirect effect	−0.2052	0.0174	−0.2393, −0.1708	−0.1683	0.0266	−0.2227, −0.1181	−0.2182	0.0207	−0.2593, −0.1774
Indirect 1	−0.1148	0.0187	−0.1524, −0.0795	−0.1417	0.0291	−0.2037, −0.0873	−0.0908	0.0234	−0.1387, −0.0464
Indirect 2	−0.0101	0.0049	−0.0204, −0.0010	−0.0031	0.0041	−0.0134, 0.0030	−0.0195	0.0088	−0.0380, −0.0029
Indirect 3	−0.0802	0.0144	−0.1088, −0.0527	−0.0234	0.0216	−0.0656, 0.0183	−0.1079	0.0171	−0.1422, −0.0750

## Discussion

The results of this study demonstrate that there is no significant difference in academic burnout between male and female college students, which aligns with existing studies reporting that academic burnout is not influenced by gender (Lin and Huang, 2014; May et al., 2015). In this study, we found that physical exercise was significantly and negatively associated with academic burnout and its three subfactors (emotional exhaustion, improper behavior, and low personal achievement) in a sample of college students, thus supporting H1. Thus, we provide evidence that physical exercise has a direct and negative predictive effect on academic burnout in this context. Previous research showed that physical exercise had the potential to not only reduce stress in college students (Herbert et al., 2020) but also improve their cognitive abilities and academic performance (Voss et al., 2011; Li et al., 2017). These factors are important in addressing academic burnout and its negative impact (Lin and Huang, 2014; May et al., 2015). Regarding the effects derived from different levels of physical exercise, our analyses showed significant differences in academic burnout levels among high, medium, and low physical exercise groups. Simultaneously, the level of academic burnout sequentially increased with decreased levels of exercise; this corresponds with the findings of previous studies (Gerber et al., 2015; Khosravi et al., 2020). This may be because more physically active university students have more benefits associated with lower academic burnout, such as better sleep quality, greater self-control, and lower physical vulnerability to stress (Seibert et al., 2016; Boat and Cooper, 2019; Lee et al., 2020; Khosravi, 2021). In addition, we found significant differences in the relationship between physical exercise level and academic burnout among college students of different genders. Thus, these results highlight the importance of considering the volume of physical exercise and gender differences when developing physical exercise programs, wherein moderate and higher levels may more efficiently alleviate academic burnout.

In addition to the direct effect, we tested for indirect effects in the relationship between physical exercise and academic burnout. We thus found that self-efficacy mediated the relationship between physical exercise and academic burnout, which supported H2. On the one hand, physical exercise helps to create positive body image for university students (Toselli and Spiga, 2017), which enhances their body satisfaction and self-efficacy (Ouyang et al., 2020). On the other hand, students with high self-efficacy tend to adopt more advantageous learning strategies and goals to improve academic performance (Alhadabi and Karpinski, 2020), which is beneficial to reducing the risk of academic burnout (Rahmati, 2015; Merhi et al., 2018). In this regard, physical exercise can influence self-efficacy, which then influences academic burnout. Given the general lack of research in this area, our results further reinforce the verified relationship between these factors. Thus, self-efficacy works as a bridge between physical exercise and academic burnout. Campus administrators should introduce programs to help students develop self-efficacy *via* physical exercise to combat academic burnout more effectively.

According to indirect pathway 2, in the total sample and female sample, resilience mediates the impacts of physical exercise on academic burnout. Therefore, it can be inferred that H3 is correct. Our findings support previous research findings that physical exercise promotes resilience in college students (Román-Mata et al., 2020; Dunston et al., 2022). Some researchers believe this is a result of physical exercise providing an appropriate setting for improving resilience (Xu et al., 2021). In addition, our study revealed that resilience was negatively associated with academic burnout. A previous study also found that the majority of those with higher levels of resilience showed lower levels of academic burnout, which was attributed the fact that resilience is a psychologically protective resource that allows students to resist the stress of school (Fernández-Castillo and Fernández-Prados, 2021). However, physical exercise in the male sample was not a significant predictor of resilience, possibly because males are more susceptible to masculinity-seeking and interpersonal relationship resolution in sports, thus affecting the resilience-promoting effect of physical exercise (Overholt and Ewert, 2015). The predictive effect of resilience on academic burnout was also not significant in the male sample, possibly because males have higher cynicism relative to females, which correlates less with resilience than emotional exhaustion and low achievement, resulting in a poor resistance of resilience to academic burnout (Isabel Rios-Risquez et al., 2016; García-Izquierdo et al., 2018). Therefore, strategies to intervene in resilience and academic burnout through physical exercise seem to be more applicable to female college students, compared to male college students.

Looking at indirect pathway 3, self-efficacy and resilience play a serial mediating role in the relationship between physical exercise and academic burnout, which supports H4. Although there is no previous direct evidence of the relationship between physical exercise, self-efficacy, resilience, and academic burnout, some studies have confirmed the role of self-efficacy in promoting resilience (Hamill, 2003; Cassidy, 2015; Sabouripour et al., 2021). Similarly, self-efficacy is an important personal resource for the development of resilience (Benard, 2004; Narayanan and Weng Onn, 2016). In conjunction with the above, we believe that college students can enhance self-efficacy *via* physical exercise, which then promotes resilience and ultimately ease their academic burnout. In addition, given the low effect size of physical exercise on resilience found in indirect path 2, we believe that self-efficacy functions as a bridge between physical exercise and resilience. In a word, physical exercise not only directly affects academic burnout among college students, but also indirectly through the serial-mediating effects of self-efficacy and resilience. This provides an important practical reference for solving the problem of academic burnout among college students.

### Limitations and future research

Although our study deepens the scholarly understanding of the relationship between physical exercise and academic burnout in college students, there were also some limitations. First, this study did not cover important factors that influence academic burnout, such as socioeconomic status, age, and personality traits (e.g., novelty seeking). Future research should consider external environmental and individual characteristic factors to reveal a more comprehensive picture of the relationship between physical exercise and academic burnout among college students. Second, the survey sample only involved students from universities in Chongqing and had a higher proportion of female students, which may reduce the generalizability and analytical efficacy of the study results. Future studies should expand the sampling scope and focus on a balanced gender ratio. Third, although we inferred the temporal ordering of the structure presentation in the model based on the positive psychological effects of physical exercise, it is not appropriate to consider any observed association as causal, given the cross-sectional design. In addition, we measured participants’ retrospective independent variable status, which may lead to reporting bias. Future research should conduct longitudinal intervention trials to measure physical exercise and academic burnout using more objective physiological and psychological indicators and focus on the characteristics of long-term changes in the relationship between the two.

### Conclusion

This study demonstrates that physical exercise has a negative impact on academic burnout and its subfactors. In this study, self-efficacy and resilience played mediating roles in the effect of physical exercise on academic burnout. College students in the high physical exercise group showed lower levels of academic burnout than those in the moderate and low physical exercise groups. We suggest that directing university students to actively engage in physical exercise for developing self-efficacy and resilience may effectively contribute to preventing academic burnout.

## Data availability statement

The original contributions presented in the study are included in the article/Supplementary material, further inquiries can be directed to the corresponding author.

## Ethics statement

The studies involving human participants were reviewed and approved by the Ethics Committee at South West University. Written informed consent from the participant’s legal guardian/next of kin is not required to participate in this study with the national legislation and the institutional requirements.

## Author contributions

KC and LG designed the study and revised the manuscript. KC and FL provided the original manuscript. LM and PZ collected and analyzed the data. All authors contributed to the article and approved the submitted version.

## Funding

This study was supported by the National Social Science Foundation of China (Title: Intervention Study of Adaptive Physical Education on the Cognitive Function of Children with Intellectual Disabilities. Number of approval: 18BTY094).

## Conflict of interest

The authors declare that the research was conducted in the absence of any commercial or financial relationships that could be construed as a potential conflict of interest.

## Publisher’s note

All claims expressed in this article are solely those of the authors and do not necessarily represent those of their affiliated organizations, or those of the publisher, the editors and the reviewers. Any product that may be evaluated in this article, or claim that may be made by its manufacturer, is not guaranteed or endorsed by the publisher.
