# Supplementary material for: How physical exercise impacts academic burnout in college students: The mediating effects of self-efficacy and resilience
Source: Front Psychol. 2022 Nov 11;13:964169. doi: 10.3389/fpsyg.2022.964169 (PMC9691659; doi:10.3389/fpsyg.2022.964169)
Supplement: Supplementary file 2 [file Data_Sheet_1.PDF]

Physical Activity Rating Scale (PARS-3)

1. What kind of physical exercise do you do regularly?

○ Light exercise (e.g. walking, radio exercises, etc.)

○ Low-intensity, less stressful exercise (e.g. recreational volleyball, table tennis, jogging, etc.)

○ More vigorous sustained exercise of moderate intensity (e.g. cycling, running, etc.)

○ Heavy, but not sustained, exercise with shortness of breath and a lot of sweating (e.g. badminton, basketball, tennis, football, etc.)

○ High-intensity sustained exercise with shortness of breath and a lot of sweating (e.g. racing, aerobics sets, swimming, etc.)

2. How many minutes at a time do you perform the above intensity physical exercise?

○ Under 10 minutes

○ 11 to 20 minutes

○ 21 to 30 minutes

○ 31 to 59 minutes

○ 60 minutes or more

3. How many times a month do you do the above-mentioned physical exercise?

○ Less than 1 time a month

○ 2 to 3 times a month

○ 1 to 2 times a week

○ 3 to 5 times a week

○ About 1 time a day

General Self-Efficacy Scale (GSES)

1. I can always solve problems if I do my best
2. I can get what I want even when people are against me
3. It's easy for me to stick to my ideals and reach my goals
4. I am confident that I can deal effectively with anything that comes my way
5. With my talent, I can handle the unexpected
6. If I put in the necessary effort, I will be able to solve most of the puzzles
7. I can face difficulties calmly because I trust my ability to deal with them
8. When faced with a difficult problem, I can usually find several solutions
9. When there's trouble, I can usually think of a few ways to deal with it
10. No matter what happens to me, I can handle it

Connor-Davidson Resilience Scale (CD-RISC)

1. I am able to adapt when changes occur
2. I have at least one close and safe person to help me when under pressure
3. Sometimes fate or God can help me when I can't get a clear solution to my problems
4. Whatever happens on my path, I can handle it
5. Past successes have given me the confidence to tackle new challenges and difficulties
6. When faced with a problem, I try to look at the humorous side of things
7. I am a stronger person because of the trials and tribulations I have gone through
8. I can easily recover after illness, injury or suffering
9. For better or for worse, I believe that everything happens for a reason
10. Whatever the outcome, I will do my best
11. Despite the obstacles, I believe I can achieve my goals
12. I don't give up even though it seems hopeless
13. When stress or crisis comes, I know where to get help
14. Under pressure, I am able to think mentally
15. I prefer to take the lead in solving problems myself rather than letting others decide the big picture
16. I will not be easily defeated by failure
17. I think I am a strong person when it comes to dealing with the challenges and difficulties in life
18. I will make an unpopular or difficult decision to influence someone if I have to
19. I can deal with unpleasant or painful feelings, such as sadness, fear and anger
20. Sometimes I have to go with my gut when dealing with difficult life situations
21. I have clear goals in my life
22. I feel in control of my life
23. I love a challenge
24. I will strive to reach my goals no matter what obstacles I encounter along the way
25. I am proud of what I have achieved

Learning Burnout Scale for College Students

1. I have my own learning methods and plans and can put them into practice
2. I feel that what I have learnt is useless
3. Expertise is easy for me to grasp
4. Getting up early in the morning and feeling tired at the thought of facing a day of study
5. It's hard for me to stay enthusiastic about learning for long
6. I am able to deal with my emotional problems calmly when studying
7. I felt exhausted after a long day of studying
8. So far, my university studies have enabled me to showcase my abilities
9. I am bored with my studies
10. I rarely study after class
11. I can do university courses
12. I often doze off when I study
13. I am very interested in my profession
14. I don't think I have enough patience when it comes to learning
15. It was easy for me to get my diploma
16. I only read when I have exams
17. I want to study but I find it boring
18. I have a lot of energy when I study
19. I rarely plan my study time
20. Exams always bore me
